# ATMP Environmental Exposure Assessment in European Healthcare Settings: A Systematic Review of the Literature

**DOI:** 10.3389/fmed.2021.713047

**Published:** 2021-12-01

**Authors:** Margaux Damerval, Christine Fagnoni-Legat, Aurélien Louvrier, Sarah Fischer, Samuel Limat, Anne-Laure Clairet, Virginie Nerich, Isabelle Madelaine, Marie Kroemer

**Affiliations:** ^1^Department of Pharmacy, University Hospital of Besançon, Besançon, France; ^2^Host-Graft Interactions Lab – Tumor - Cell and Tissue engineering (UMR 1098 INSERM/UFC/EFS), University of Franche-Comté, Besançon, France; ^3^Department of Oral and Maxillofacial Surgery, University Hospital of Besançon, Besançon, France; ^4^Department of Pharmacy, APHP, Hôpital Saint-Louis, Paris, France

**Keywords:** advanced therapy medicinal products (ATMP), cell and gene therapy (CGT), environmental exposure, environmental shedding, cellular therapy, healthcare settings

## Abstract

Since 2007, a new class of biologic products for human use called “advanced therapy medicinal products (ATMP)” have been legally integrated in the European Medical Agency. They consist of recombinant nucleic acid, engineered cells, cells, or tissues. In the United States, ATMP fall under the regulatory framework of biological products and the term “cell and gene therapy product” is used in the legislative and regulatory documents. Potential clinical applications are broad, particularly, in the field of cancer, inherited genetic disease, and regenerative medicine. Indeed, the benefit conferred by CD19 chimeric antigen receptor T cells led to the first engineered cell therapy products to be approved by the Food and Drug Administration (FDA) in 2017. Gene therapy products to treat orphan diseases are also extensively developed with many clinical trials ongoing in the world. Nevertheless, the use of these therapeutic products is complex and requires careful considerations in the terms of regulatory and hospital setting requirements, such as storage, handling, administration, and disposal which justify the implementation of a secured medication circuit. Through this systematic review of the literature, the authors wanted to compile data on the assessment of environmental exposure related to the use of ATMP in healthcare setting to secure their medication circuit. A literature search was conducted on PubMed and Web of Science, and 32 publications dealing with environmental exposure assessment and ATMP were selected. In addition, marketed ATMPs were identified and data regarding the environmental concerns were extracted from product information sections from European Public Assessment Reports (EPAR). The environmental contamination assessments were mainly addressed in the reviews rather than in original articles related to the use of ATMP. Most of the product information sections from EPAR suggested precautions rather than requirements when dealing with environmental consideration following ATMP handling. Nevertheless, these precautions usually remain elusive especially concerning waste disposal and the detection of biological material on the work surfaces, and mainly relate to the genetically modified organisms (GMO) over non-GMO cellular products. Pharmaceutical oversight and adherence to the good preparation practices and good clinical practices are essential to ensure the safe use in term of environmental concern of these new therapeutic products in healthcare setting.

## Introduction

Since 2007, a new class of biological products for human use called advanced therapy medicinal products (ATMP) are legally integrated in the European Medicines Agency (EMA) (1). These innovative biotechnological products consist of recombinant nucleic acids and engineered cells or tissues which are at the origin of the complexity of their pre-clinical and clinical development, handling, regulatory framework, and classification (2). In Europe, ATMP are divided into four subcategories known as somatic cell therapy medicinal products, gene therapy medicinal products, tissue engineered products, and combined ATMP (3). In the United States (US), ATMPs also fall under the regulatory framework of biological products but only encompass two subcategories called cellular and gene therapy (CGT) products (2). Indeed, the term “CGT product” is the one used in the US legislative and regulatory documents (4). Potential clinical applications of ATMP are broad, particularly in the field of cancer, inherited genetic diseases, and regenerative medicine (5, 6). More importantly, the use of these products is rapidly expanding in the clinical settings and sometimes they are used as last resort when conventional therapeutic approaches are ineffective (7, 8).

The successful clinical transition from bench to bedside of cellular and gene therapies in the early 2000s led to the start of early phase clinical trials (9, 10). Since, several gene and gene-modified cell-based therapies are approved by the Food and Drug Administration (FDA) and EMA. Imlygic^®^ (talimogene laherparepvec, T-VEC), a genetically modified oncolytic vector, was the second gene therapy product approved in Europe in 2016 (5, 11). Then, the clinical benefit conferred by the CD19 chimeric antigen receptor (CAR)-T cells, led to the first engineered cell therapy products to be approved by the FDA in 2017 (8, 12). Pivotal studies showed a high rate of durable responses and an increase in the global survival despite high grade toxicities (8, 13, 14). These breakthrough in the field of cancer medicine prompted to the clinical development of CAR-T cells for other hematological malignancies, such as multiple myeloma and solid tumors, such as glioblastoma despite their immunosuppressive microenvironment and technological barriers preventing T-cell entry (15, 16). Beyond their successful development in the field of immuno-oncology, ATMP are currently extensively developed in orphan diseases addressing the unmet medical needs (17, 18). Another viral vector, Zolgensma^®^ (onasemnogene abeparvovec), developed in the orphan disease spinal muscular atrophy proved its effectiveness in terms of overall survival, motor function, motor milestone achievements, and motor unit function (19). Furthermore, the development of cellular therapy products is illustrated by the approval of several ATMP in various diseases, such as limbal stem cell deficiency with Holoclar^®^ (*ex vivo* expanded autologous human corneal epithelial cells containing stem cells), perianal fistulas in Crohn's disease with Alofisel^®^ (Darvadstrocel), and cartilage defect in the knee with Spherox^®^ (spheroids of human autologous matrix associated chondrocytes) (20–22). One common point between the studies involving ATMP is the lack of information concerning the assessment of environmental exposure. The use of these therapeutic products is complex and requires careful considerations in terms of regulatory and hospital setting requirements, such as storage, handling, administration, and disposal which justify the implementation of a secured medication circuit. In the framework of ATMP, the environmental risks are described mainly as the risk of transmission of the gene modified organisms to humans other than the patient, to animals or to the environment at large (23). In Europe, the marketing authorization of ATMP falls under the mandatory scope of a central authorization procedure. Among the data submitted by the developer, an environmental risk assessment (ERA) must be present. The specific guidelines dedicated to genetically modified organism (GMO) (EMEA/CHMP/BWP/473191/2006) for both the clinical trials and marketing authorization have recently been reviewed by Whomsley R. and colleagues (24). ERA for GMO should include six steps that are: (1) the identification of characteristics which may cause adverse effects, (2) the evaluation of the potential consequences of each adverse effect if it occurs, and of the magnitude of each identified consequence, (3) the evaluation of the likelihood of the occurrence of each identified potential adverse effect, (4) the estimation of the risk posed by each identified characteristic of the GMO, (5) the application of management strategies covering the risks from the marketing of the GMO, and (6) the determination of the overall risk of the GMO. These steps should be transposable to the cell therapy medicinal products. Routes through which ATMP could come in contact with the human beings other than the intended patient, or enter the environment, include dispersal of portions of product during normal handling and use; accidental dissemination during handling and use; disposal of unused or waste medicinal product; and dispersal of GMO containing patient excreta. Once released, the GMO may spread, undergo genetic or phenotypic change, compete with existing species, infect tissue, remain latent, reproduce, transfer genetic material to other micro-organisms, transfer genetic material to human beings, animal, or plant species, and degrade. Despite the necessity of ERA in both the clinical trials and marketing authorization, environmental exposure assessment related to ATMP handling in healthcare setting, notably in pharmacy preparation unit dedicated or not to their manipulation, deserves to be considered. Because of the heterogeneity of ATMP, it is difficult to define the general requirements for environmental exposure assessments that are applicable to all of them, apart from a dichotomous classification between the somatic cell therapy and gene therapy medicinal products.

Through this systematic review of the literature, the authors wanted to compile the data on environmental exposure assessment related to ATMP use especially in the healthcare settings to secure their medication circuit.

## Materials and Methods

### Eligibility Criteria

The population, interventions, comparison, and outcomes (PICO) model was used to formulate the questions for this study: (1) studies that considered environmental exposure assessment related to ATMP use (population), (2) studies dealing with the description of environmental exposure assessment related to ATMP use (interventions), (3) comparison criteria was not applicable, (4) studies that reported how to prevent environmental exposure in the use of ATMP and if there is a risk or not (outcomes).

### Search Strategies

We searched Pubmed/Medline and Web of science databases for the studies published from January 1, 2000 to March 31, 2021. Selected keywords and Medical Subject Heading (MeSH) terms were individually selected by means of the National Library of Medicine controlled vocabulary thesaurus used for indexing articles for PubMed. The keywords and MeSH terms were combined to conduct the literature search as described in Table 1. This study was conducted and reported according to the Preferred Reporting Items for Systematic Reviews and Meta-Analyses (PRISMA) guidelines (25).

**Table 1:** The keywords and MeSH terms to conduct the literature search.

**Databases**	**Research equations**
Pubmed	•(((((((((“Environmental Exposure”[Mesh]) AND “Biological Therapy”[Mesh]) NOT “Blood Patch, Epidural”[Mesh]) NOT “Blood Transfusion”[Mesh]) NOT “Cytapheresis”[Mesh]) NOT “Fecal Microbiota Transplantation”[Mesh]) NOT “Hematopoietic Stem Cell Mobilization”[Mesh]) NOT “Immunomagnetic Separation”[Mesh]) NOT “Immunomodulation”[Mesh]) NOT “Organotherapy”[Mesh] •shedding risk assessment AND gene therapy •shedding risk assessment AND cellular therapy •shedding risk assessment AND oncolytic virus •environmental shedding AND gene therapy •environmental shedding AND cellular therapy •environmental shedding AND oncolytic virus •environmental exposure AND advanced medicinal therapeutic product •safety AND advanced medicinal therapeutic product
Web of science	•TS = (risk assessment^*^ AND gene therapy AND cell therapy product^*^) •TS = (environmental risk assessment^*^ AND cell therapy product^*^) •TS = (environmental risk assessment^*^ AND gene therapy^*^) •TS = (environmental risk assessment^*^ AND advanced therapy medicinal product^*^) •TS = (environmental shedding^*^ AND gene therapy^*^) •TS = (environmental shedding^*^ AND cell therapy product^*^) •TS = (environmental contamination^*^ AND gene therapy^*^) •TS = (health risks^*^ AND gene therapy^*^ AND cellular therapy product^*^) •TS = (risks management^*^ AND gene therapy^*^ AND Cellular therapy product^*^) •TS = (risks management^*^ AND advanced therapy medicinal product^*^)

### Inclusion and Exclusion Criteria

Article reporting environmental concerns related to the use of ATMP in healthcare setting written in English or French were included in this review. Exclusion criteria included translational research using the cells or animals and congress poster.

### Study Selection and Data Extraction

The articles were submitted to a public reference manager (Zotero^®^ software) to eliminate the repeated articles. Then, possible relevant articles were screened using the title and abstract by two reviewers (MK and MD) and articles that did not meet the inclusion criteria were excluded. Subsequently, the remaining full-text articles were examined by two reviewers (MK and MD). Any disagreement was resolved through discussion until a consensus was reached. The following items were extracted from each full text article that met the inclusion criteria, if available: year of publication, journal type, main location of first author, sponsor, conflicts of interest (yes, no, and not reported), type of article (original research or literature review), type of ATMP (cellular, gene therapy, and both), aim of the paper, examination of environmental exposure assessment (production, preparation, disposal), what's being watched (ATMP handling, excreta, and not reported), technique use to assess environmental exposure (PCR, sequencing, monitoring, and not reported), regulatory framework (yes or no), how to avoid environmental exposure (quarantined treated patients, disinfectants/decontamination, hygiene measures, sterilization, and not reported), and environmental risk (yes, potential, and not reported). In addition, marketed ATMP were identified and data dealing with environmental concerns were extracted from product information sections from the European Public Assessment Reports (EPAR).

### Risk of Bias Assessment

Two reviewers (MK and MD) independently assessed the methodological quality of articles. The selected articles were categorized into three groups: relevant, irrelevant, and unsure. The articles categorized as irrelevant by both the reviewers were eliminated from the study. Second, the full text of each selected article was independently analyzed by both the reviewers that make a list of articles to be included. The two list were compared, and a consensus was found in the case of disagreements between the two reviewers. When an agreement was not reached, a third reviewer made the final decision. The main reason for each article exclusion was recorded.

Additionally, the reference lists of all the selected articles were screened to identify other potentially relevant articles that were not identified by means of the electronic source. Pivotal studies of ATMP actually and previously marketed in Europe and their product information sections from EPAR were screened and analyzed.

## Results

### Article Selection

**Figure 1:**
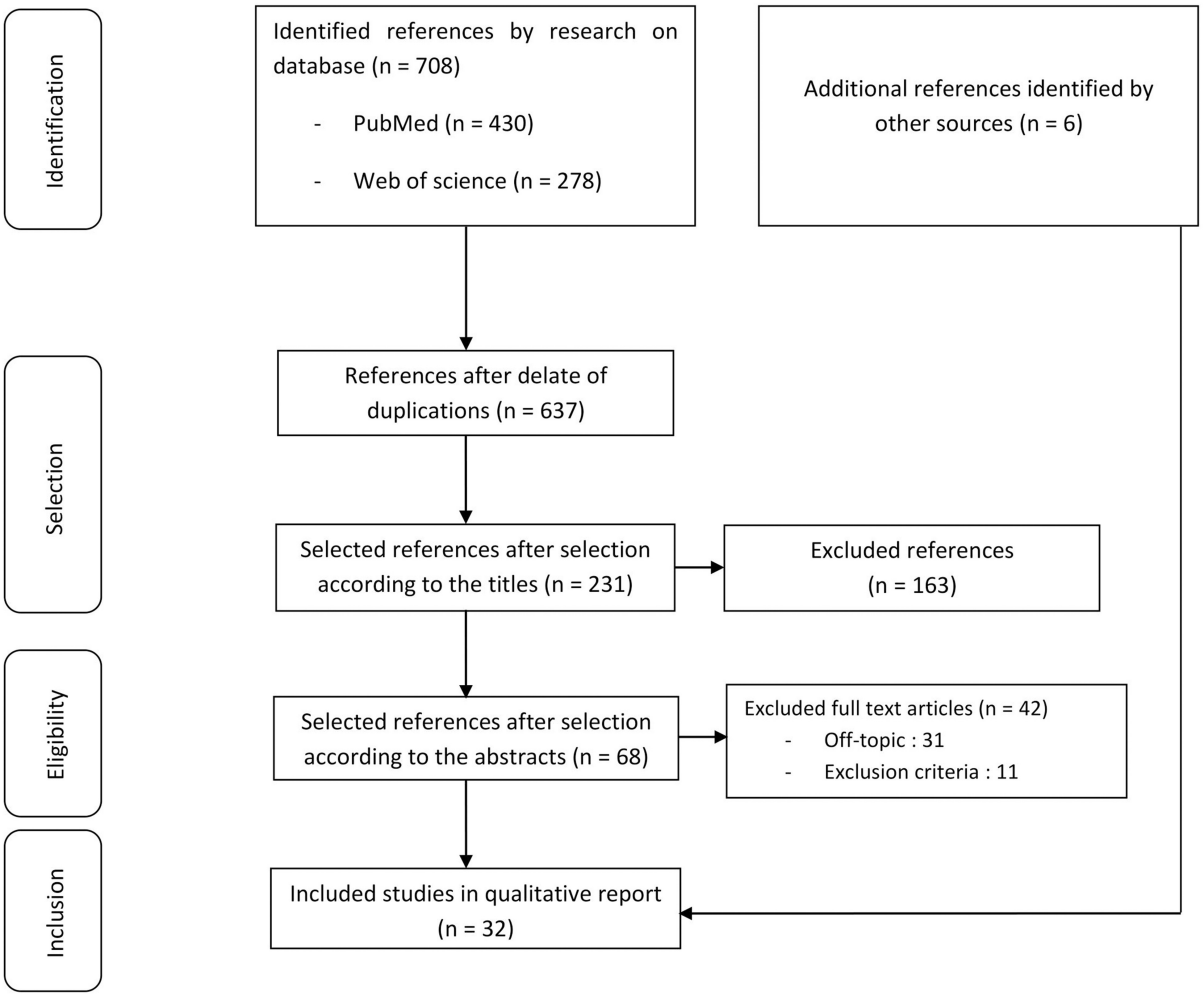
Preferred Reporting Items for Systematic Reviews and Meta-Analyses (PRISMA) flowchart for the study selection.

The literature search conducted on PubMed and Web of Science identified 708 articles, among which 71 were duplicate articles and 569 were excluded after reviewing the titles and abstracts that did not match the eligibility criteria (Figure 1). A total of 68 articles were included for full text review, among which 42 were excluded because they were off topic, or they did not match the eligibility criteria. Two ATMP pivotal studies have been added (26, 27). Overall, 32 articles were eligible for the present systematic review.

### Characteristics of Selected Articles

The characteristics of the 32 selected articles are summarized in Table 2. Few of them were published in the early 2000s, and there has been a considerable increase in the published articles ever since 2010 (75%, *n* = 24). Only four articles were original research, not including pivotal studies (28–31). Other were mainly state of the art or literature review.

**Table 2:** Synthesis of basic elements of 32 included articles.

	**Number**	**Percentage**
**Years of publication**		
2000–2009	8	25
2010–2019	24	75
**Main location of the authors**		
University	9	28
University and hospital	3	9
Hospital	9	28
Other	11	35
**Original research**		
Yes	6	19
No	26	81
**Literature review**		
Yes	26	81
No	6	19
**Conflict of interest**		
Yes	8	25
No	18	56
Not reported	6	19
**ATMP**		
Cellular	1	3
Gene therapy	24	75
Both	7	22
**Examination of environmental exposure assessment**		
Production	2	5
Preparation	12	31
Disposal	25	64
**What's being watching**		
ATMP handling	14	44
Excreta	13	41
Not reported	5	15
**Technique use to assess environmental exposure**		
PCR, sequencing	14	41
Monitoring	9	27
Not reported	11	32
**Regulatory framework**		
Yes	15	47
No	17	53
**How to prevent environmental exposure**		
Quarantine treated patients	8	16
Disinfectants/Decontamination	16	33
Hygiene measures/PPE	8	16
Sterilization	5	10
Not reported	12	25
**Environmental risk**		
Yes	7	22
Potential	20	63
Not reported	5	15

*PCR, polymerase chain reaction; PPE, personal protective equipment*.

### Synthesis of the Basic Elements of Selected Article Dealing With ATMP Environmental Exposure Assessment

The synthesis of basic elements from the 32 selected articles is summarized in Table 3. Among the selected articles, 75% (*n* = 24) deal with gene therapy and 3% (*n* = 1) with cellular therapy. Remaining articles concerned ATMP regardless of their classification. Among the selected articles, environmental exposure assessment related to ATMP was examined during manufacturing (5%, *n* = 2), handling and manipulation (31%, *n* = 12), and waste disposal (64%, *n* = 25).

**Table 3:** The characteristics of the 32 selected articles.

**Reference**	**Year**	**Journal**	**Main location of first author**	**0 = original research1 = litterature review**	**1 = cellular therapy 2 = gene therapy 3 = both**	**Aims of the paper**	**Examination of environnemental shedding 1 = manufacturing 2 = preparation (PUI) 3 = after use**	**What's being watched ?**	**Technique use to detect**	**Regulatory framework**	**How to avoid environmental risk ?**	**Environmental risk**
Bachtarzi et al. (32)	2019	Clinical	University	1	3	To compare control use of GMO in Europe, USA and Japan.	3	Excreta	PCR	Yes	Quarantine treated patient	Yes
Iglesias-Lopez et al. (33)	2019	Clinical	University	1	3	To summarize regulatory data about GMO in Europe and USA.	2, 3	Excreta	PCR	Yes	Physical, chemical and biological barriers	Not reported
Bubela et al. (34)	2019	Clinical	University	1	3	To review regulatory data for GMO in Canada.	3	Excreta	Environmental safety monitoring	No	Not reported	Yes
Pinturaud et al. (35)	2018	Clinical	Hospital	1	3	To identify the role of the pharmacist in the system of advanced therapy medicine.	2	ATMP handling	Not reported	Yes	Not reported	Yes
Sharpe (36)	2018	Clinical	Hospital	1	2	To review gene therapy products showing safety strategies.	3	ATMP handling	PCR	No	Not reported	Yes
Okeke et al. (37)	2017	Clinical	Hospital	1	2	To review the caracteristics of Ankara virus used as a vector.	3	ATMP handling	Not reported	Yes	Disinfectants	Yes
Renner et al. (38)	2015	Clinical	Hospital	1	3	To summarize regulatory data about GMO in Germany.	3	Excreta	PCR Environmental safety monitoring	Yes	Hygiene measures Decontamination	Potential
Montemurro et al. (39)	2015	Clinical	Hospital	1	1	To describe the italian approach concerning the arrival of the advanced medicinal products in an hospital.	1, 2	ATMP handling	Not reported	Yes	Hygiene measures Decontamination	Not reported
Lucas-Samuel et al. (40)	2015	Clinical	ANSM	1	3	To describe french regulatory data concerning advanced medicinal products.	1, 2, 3	ATMP handling	Environmental safety monitoring	Yes	Hygiene measures Decontamination	Potential
Buijs et al. (41)	2015	Clinical	Hospital & University	1	2	To review preclinical and clinical development about oncolytic viruses.	3	Excreta	Environmental safety monitoring	No	Not reported	Yes
Narayanan et al. (42)	2014	Clinical	Institut	1	2	To review points of view of differents actors of gene therapy.	3	Excreta	Environmental safety monitoring	No	Not reported	Potential
van den Akker et al. (43)	2013	Clinical	Institut	1	2	To present the methodology to do an ERA.	3	/	Environmental safety monitoring	No	Quarantine treated patient	Potential
Hoeben et al. (44)	2013	Clinical	University	1	2	To summarize the potential biological risks of oncolytic viruses vectors.	3	/	Environmental safety monitoring	No	Quarantine treated patient	Potential
Goossens et al. (45)	2013	Clinical	Institut	1	2	To present how identify risks with Ankara viruses.	2, 3	ATMP handling	PCR	No	Hygiene measures Decontamination Quarantine treated patient Ethanol	Potential
Baldo et al. (28)	2013	Clinical	Institut	0	2	To present an ERA.	2, 3	ATMP handling	PCR, biological assay	Yes	Hygiene measures Decontamination Quarantine treated patient Autoclaving	Potential
Verheust et al. (46)	2012	Clinical	Institut	1	2	To pick up characteristics of Ankara viruses as a vector and discuss about its safety.	2, 3	Excreta	PCR, biological assay	Yes	Cleaning up the skin with alcohol 70 % Hygiene measures Chemical decontamination, steam sterilization Quarantine treated patient	Potential
Koppers-Lalic and Hoeben (47)	2011	Clinical	University	1	2	To classify viruses according to their environmental impact.	3	/	Not reported	No	Not reported	Potential
Tiesjema et al. (48)	2010	Clinical	Institut	1	2	To present shedding data of viral vector according to the route of administration.	3	Excreta	PCR, southern blot, ELISA, transgene expression, infectious assay	No	Not reported	Potential
Brandon et al. (49)	2010	Clinical	Institut	1	2	To present shedding data of viral vector according to the route of administration.	3	Excreta	PCR, southern blot, ELISA, transgene expression, infectious assay	No	Not reported	Potential
Anliker et al. (29)	2010	Clinical	Institut	0	2	How do an ERA ?	3	ATMP handling	Not reported	Yes	Hygiene measures Decontamination Quarantine treated patient	Potential
Pauwels et al. (50)	2009	Clinical	Institut	1	2	To review how to improve the safety with the use of Lentivirus vector.	2	ATMP handling	Not reported	No	Hygiene measures Decontamination Disinfectants PPE	Not reported
Kuhler et al. (51)	2009	Clinical	Medical product agency	1	3	To discuss about environmental impact of biological medicinal products.	3	ATMP handling	Not reported	Yes	Disinfectants	Not reported
Schenk-Braat et al. (52)	2007	Clinical	Hospital	1	2	To review studies about gene therapy and environmental impact.	3	Excreta	PCR, biological assay ELISA Environmental safety monitoring	No	Not reported	Potential
Bleijs et al. (53)	2007	Clinical	University	1	2	To summarize regulatory data concerning gene therapy in the Netherlands.	3	/	Not reported	Yes	Quarantine treated patient	Potential
Moss et al. (31)	2004	Clinical	University	0	2	Clinical study about safety of a viral vector used in cystic fibrosis.	3	Excreta	PCR	No	Not reported	Potential
Tenenbaum et al. (54)	2003	Clinical	Hospital & University	1	2	To review characteristics of two viral vectors.	3	Excreta	PCR	No	Cleaning up materials with alkaline solutions with a pH greater than 9 or by autoclaving	Potential
Gaudet et al. (26)	2013	Clinical	University & Hospital	0	2	To show the efficacy and tolerability of the product.	3	Excreta	PCR	No	Not reported	Yes
Thompson et al. (27)	2018	Clinical	Hospital	0	2	To show the efficacy of the product.	3	/	Environmental safety monitoring	No	Not reported	Not reported
McBride et al. (30)	2018	Clinical	University	0	2	To provide an overview of the preparation and handling of imlygic.	2	ATMP handling	PCR	Yes	PPE Occlusive bandage Disinfectants: bleach, isopropanol	Potential
Petrich et al. (55)	2020	Pharmaceutical	Hospital	1	2	To provide a comprehensive review of gene therapy.	2	ATMP handlig	Not reported	No	Disinfectants	Potential
Stoner et al. (56)	2003	Pharmaceutical	Hospital	1	2	To illustrate the development of procedures to minimize risks to health, patient safety and the environment.	2	ATMP handling	Not reported	Yes	Disinfectants PPE Decontamination Autoclaving, incineration Occlusive bandage	Potential
Vulto et al. (57)	2007	Pharmaceutical	University	1	2	To specify the requirements of each step for the gene therapy drug circuit.	2	ATMP handling	Not reported	Yes	PPE Decontamination, Disinfectants Autoclaving, inactivation	Potential

Almost half of the articles (47%, *n* = 15) involve regulatory framework, such as good manufacturing practices, good preparation practices, and/or European Union (EU) legislation. PCR was used as a main technique to detect ATMP on work surfaces and the excreta or blood of a patient (26, 28, 31–33, 36, 38, 45, 46, 48, 49, 52, 54). The follow-up of a patient after treatment was approached by nine articles to evaluate the potential risk of dissemination of the ATMP (27, 34, 38, 40–44, 52). Yet, different ways of disposal were described to prevent the environmental shedding and quarantine of patients was mainly proposed (*n* = 8; 16%). Hygiene measures using the disinfectants to decontaminate and clean work surfaces and equipment as sodium hypochlorite, ethanol, or alkaline solutions following the use of ATMP were described (28–30, 33, 37–40, 45, 46, 50, 51, 54–57). Waste sterilization and especially waste autoclaving was addressed within five articles (28, 46, 54, 56, 57). Excreta, such as urine and feces were tested to assess the environmental shedding of ATMP following their administration to the patients (48, 49, 52, 54). Thus, the route of ATMP administration as well as the viral vector characteristics in case of GMO might impact the environmental shedding of ATMP.

Overall, 85% of the selected articles reported a potential environmental risk of dissemination following the use of ATMP. This risk was proven (26, 32, 34–37, 41) or considered to be potential (28–31, 38, 40, 42–49, 52–57) and concerns mainly GMO.

### ATMP Product Information Sections From EPAR

Marketed ATMPs in EU are presented in Table 4. Among all EPAR studied (15/15), waste disposal following the local guidelines was recommended. Other information was found inconsistently, such as what to do in case of accidental exposure, the necessity to wear a personal protective equipment during ATMP handling, and the use of certain disinfectants after handling the clean work surfaces.

**Table 4:** Environmental exposure assessment consideration from the European Public Assessment Reports (EPAR) of advanced therapy medicinal products (ATMP) in Europe.

	**Name**	**Pharmaceutical form**	**Route of administration**	**General description**	**Therapeutic indication**	**Approved by/date**	**Stopped**	**Environmental exposure assessment consideration**
Cell therapy medicinal products	Alofisel®	Suspension for injection	Intralesional use	Human adipose tissue-derived MSCs	Complex perianal fistulas in CD	EMA 2018 March		Local requirements
	Chondrocelect®	Implantation suspension	Implantation	Autologous cell therapy based on chondrocytes	Cartilage defects	EMA 2009 October	2016 July	Local requirements
	MACI®	Implantation matrix	Implantation	Cultured chondrocytes on a porcine type I/III collagen membrane	Single or multiple symptomatic full-thickness cartilage defects of the knee with or without bone involvement in adults	EMA 2013 June	2014 September	Local requirements
	Provenge®	Dispersion for infusion	Intravenous use	PBMNCs (primarily DCs) activated with PAP and GM-CSF	Asymptomatic or minimally symptomatic metastatic castrate resistant (hormone refractory) PCA	EMA 2010 September	2015 May	Local requirementsAseptic handlingPotential transmission
	Holoclar®	Living tissue equivalent	Implantation	HCEpC containing stem cells	Severe limbal stem cell deficiency	EMA 2015 February		Any unused medicinal product or waste material must be returned to the manufacturer.
Gene therapy medicinal products	Glybera®	Solution for injection	Intramuscular use	In vivo AAV-based gene therapy	Lipoprotein lipase deficiency	EMA 2012 October	2017 October	Local requirementsVirucidal disinfectant
	Imlygic®	Solution for injection	Intralesional use	Live, attenuated HSV-1 genetically modified to express hGM-CSF	Unresectable cutaneous, subcutaneous, and nodal lesions in recurrent melanoma after initial surgery	EMA 2015 December		Local requirementsPPEAccidental exposureOcclusive bandage
	Luxturna®	Solution for injection	Subretinal use	Live, non-replicatingAAV2 genetically modified to express hRPE65 gene	Biallelic RPE65 mutation-associated retinal dystrophy	EMA 2018 November		Local requirementsPPEVirucidal disinfectantAccidental exposure
	Kymriah®	Dispersion for infusion	Intravenous use	CD19- targeted genetically modified T-lymphocytes	Patients up to 25 years of age with refractory B-ALL, who are in relapse post-transplantation or in second or later relapse, and adult patients with r/r DLBCL after two or more lines of systemic therapy	EMA 2018 August		Local requirementsPPE
	Yescarta®	Dispersion for infusion	Intravenous use	CD19-targeted genetically modified T- lymphocytes	Adult patients with r/r DLBCL and PMBCL after 2 or more lines of systemic therapy	EMA 2018 August		Local requirements
	Zalmoxis®	Dispersion for infusion	Intravenous use	Genetically modifiedT-lymphocyte with a retroviral vector encoding 1LNGFR and HSV-TK	Haploidentical-HSCT adult patients with high-risk hematological malignancies	EMA 2016 August	2019 October	Local requirementsPPEAppropriate disinfectant
	Zolgensma®	Solution for infusion	Intravenous use	AAV9 vector containingfunctional copy of the SMN1 gene	Pediatric patients < 2 years of age with SMA and bi-allelic mutations in the SMN1 gene	EMA 2020 May		Local requirementsPPEAppropriate disinfectantAccidental exposureGood-hand hygiene
	Strimvelis®	Dispersion for infusion	Intravenous use	Transduced CD34 C cells with a retroviral vectorencoding human ADA	ADA-SCID	EMA 2016 May		Local requirementsAppropriate disinfectantPotential transmission
	Zynteglo®	Dispersion for infusion	Intravenous use	CD34 C cells encodingbA-T87Q-globin gene	Patients up to 12 years old with beta thalassemia who require regular blood transfusions	EMA 2019 May		Local requirementsPotential transmission
Tissue engineered products	Spherox®	Implantation suspension	Intraarticular use	Tissue spheroids of Human matrix-associatedchondrocytes	Symptomatic articular cartilage defects of the femoral condyle and the patella of the knee with defect sizes up to 10 cm2 in adults	EMA 2017 July		Local requirements

*AAV, adeno-associated virus; ADA-SCID, adenosine deaminase severe combined immunoeficiency; CD, Crohn's disease; DC, dendritic cell; DLBCL, diffuse large B-cell lymphoma; EMA, European Medicines Agency; GM-CSF, granulocyte macrophage colony stimulation factor; HCEpC, human corneal epithelial cell; HSCT, hematopoietic stem cell transplantation; hRPE, human retinal pigment epithelium; HSV, herpes simplex virus; MSC, mesenchymal stem cell; PAP, prostatic acid phosphatase; PBMNC, peripheral-blood mononuclear cell; PCa, prostate cancer; PMBCL, primary mediastinal large B-cell lymphoma; PPE, personal protective equipment; SMA, spinalmuscular atrophy; and SMN, survival motor neuron*.

## Discussion

ATMP represent a breakthrough in the field of medicines whose active substance is produced from living tissue and demonstrate the culmination of fundamental research in biotechnology. They provide the opportunity of bringing the most innovative projects coming from translational research to the clinical setting. In line with their medication status, their management in hospital depends on the pharmacies of the hospitals (58). Their complexity and technical specificity in terms of supply, reception, storage, handling, administration, and disposal imply the creation of a dedicated medication circuit. The rapidly growing area of ATMP leads to the implementation of risk minimization measures by the pharmacists to prevent environmental and occupational exposure. This work allowed to establish a state of the art of environmental exposure assessments related to the use of ATMP in healthcare settings through the analysis of both the literature and, for ATMP with marketing authorizations in Europe, their pivotal studies, and their product information section from the EPAR. Through our literature research, 32 articles dealing with the environmental risk assessment of ATMP were selected. Among the 32 articles selected, more than three quarter were published over the last 10 years, demonstrating the rapidly growing area of ATMP.

Two articles addressed the manufacturing step of ATMP in terms of regulatory framework, manufacturing, and quality control guidelines. The manufacturing step mostly focused on environmental concern related to the prevention of cross contamination and the establishment of process and standard operating procedures (SOP) to maintain a clean working environment to protect the ATMP. However, the measures taken to protect the ATMP indirectly apply to protect the environment. Twelve selected articles addressed the preparation steps of the ATMP in healthcare setting. Hygiene measures and decontamination were systematically mentioned with, among others, the use of bactericidal or virucidal agents to prevent environmental shedding. In an original article of McBride, T-VEC handling was presented (30). To prevent environmental shedding, the authors recommended the use of personal protective equipment during T-VEC preparation and administration and the use of disinfectants to clean work and room surfaces exposed to T-VEC. Similar recommendations were made by Stoner N et al. and Pietrich J et al. (55, 56). Nevertheless, the realization of a dedicated test, such as PCR to assess the presence of the GMO following its utilization and cleaning was not suggested. In 2007, Vulto AG et al. published general guidance about gene therapy handling within hospital pharmacy and suggested similar precautions should be taken in the handling of gene medicine and cytotoxic agents, especially concerning the prevention of cross-contamination (57). Unlike antineoplastic drugs, the existence of dedicated kits to assess environmental contamination on the work surfaces were not mentioned. In a study realized by Moss RB et al. that aimed to assess the safety and efficacy of a viral vector for the treatment of cystic fibrosis, the authors analyzed excreta of the patients using PCR assays to assess environmental shedding of the virus (31). Despite detection of the virus in the sputum samples of a patient, no minimal recommendations were proposed by the authors to prevent environmental shedding. Similar observations were related in the study of Baldo *et al*., which deals with gene therapy having a potential risk of dissemination depending on the vector used (28). According to the authors, it is important to analyze environmental shedding with regards to the capacity of the virus to replicate and resist within “the environment” and to quarantine the treated patients, if necessary. Environmental shedding may also depend on the route of administration of the ATMP. Indeed, Tiesjema et al. indicated that the routes of shedding for HAdV-5 depend on the route of administration (48). In the Glybera^®^ 's (alipogene tiparvovec) pivotal study, the authors specified that following its administration, the treatment may result in a low risk of dissemination in the environment (26). Indeed, the genetically modified viruses were detected in blood, urine, and saliva of the treated patients by qualitative PCR until several weeks following the ATMP injections. Nevertheless, no recommendations regarding the management of these excreta were formalized by the authors. No information concerning storage, handling, the detection of ATMP on the work surfaces and waste disposal were mentioned in original articles from our literature search except for T-VEC (30). As previously described, original research articles mainly deal with investigational medicinal product safety and efficacy.

Most of product information sections from EPAR suggest that specific precautions should be taken regarding environmental consideration following ATMP handling. Thus, in a section entitled “clinical particular,” the summary of product characteristics (SPC) of tisagenlecleucel indicates the precautions that might be taken by the healthcare professionals before handling or administering the medicinal product to prevent transmission. The precautions to be taken during transport and for wastes disposal are also mentioned in a section entitled “Special precautions for disposal and other handling.” Thus, tisagenlecleucel “should be transported within the facility in closed, break-proof, and leak-proof containers.” Waste disposal is not much discussed. Yet, it is advisable to follow the local guidelines for biological wastes disposal. Concerning axicabtagene ciloleucel, the precautions suggested were identical. Overall, there are dedicated precautions for the disposal and handling of CAR-T cells products in the SPC but no information about the risk of surface contamination and product detection were specified. In the T-VEC section “special precautions for disposal and other handling,” recommendations concerning handling and administration, personal protective equipment, accidental spills, and waste disposal were specified. The same recommendations were specified in the product section information for Luxturna^®^ (voretigene neparvovec), onasemnogene abeparvovec, Zolgensma^®^, Provenge^®^ (Sipuleucel-T), Zalmoxis^®^, and Strimvelis^®^. Nevertheless, information still remains elusive especially concerning waste disposal and the detection of medicinal product on work surfaces. Finally, we noticed that the procedure to follow in case of accidental exposure was also detailed in the same section referring to use virucidal agent in case of spill(s). As far as cell therapy is concerned, the environmental exposure assessment is once again a poorly discussed subject. Yet, among cell therapy products, only the SPC of Chondrocelect^®^ specifies, without any further details, that any drug or waste material must be disposed of in accordance with the current regulations.

Management of ATMP is complex, preventing the establishment of a single standardized pharmaceutical circuit for all of them. Furthermore, their specific storage and preparation as well as their classification as GMO or not determine how they need to be handled. Regarding GMO, the assessment of the probability that a potential hazard occurs that determines the level of risk. The level of risk then allows to determine ways to control them to ensure the protection of humans and the environment. As discussed above, antineoplastic drug handling implies a strict aseptic process to prevent cross-contamination within the pharmacy preparation units. Indeed, the experience of pharmacists, justified by the centralization of the reconstitution of antineoplastic drugs, provides an adequate basis for the handling of ATMP in health settings (55, 58, 59). Environmental concern regarding the use ATMP and not only GMO, as previously described, require adaptation in the pharmacies in terms of facilities, equipment, SOP, and waste disposal (57). Storage and manipulation of ATMP need to be performed in a dedicated area. The manipulation of GMO must be conducted in contained cabinet or isolator in negative pressure relative to the pressure of the immediate environment to protect the worker and the environment as well as the product itself. A dedicated high-efficiency particulate air (HEPA) filtering of the extracted air to protect the environment and input air to protect the product is necessary in area of both GMO and non-GMO ATMP manipulation. The establishment of SOP for storage, cleaning, preparation, personal protective clothing dedicated to preparation and administration, transport, accidental exposure, disinfection and decontamination, and disposal of waste is a minimum requirement to prevent environmental shedding. Obviously, these SOPs might be interconnected. Thus, whatever the ATMP, and considering the GMO risk group, disposable personal protective clothing, handling, and administration equipment directly in contact with the ATMP should be autoclaved (sterilization at 134°C during 20–30 min in air saturated with water vapor) if possible, using appropriate sealed container and then incinerated. Non-disposable equipment and material should be cleaned according to institutional SOP and manufacturer instruction to prevent environmental shedding. As mentioned above, the instructions present within EPAR, when they exist, always remain elusive. However, because of their diversity, GMO and especially viruses may have heterogeneous sensitivity to liquid chemical disinfectants. In that context, the recombinant associated viruses that are already used as gene delivery vehicles for approved ATMP have been described as the good virus models for testing the virucidal efficacy of disinfectants. Two studies evaluating the chemical sensitivity of different human adenovirus serotypes have concluded that the inactivation method varies according to each virus serotype demonstrating the need for knowledge and thus providing clear instructions for inactivation methods suitable for each ATMP using a viral vector (60, 61). Additionally, both studies demonstrated that complete inactivation using suitable disinfectants can be done safely and quickly.

This manuscript has several deficiencies. The articles included were limited to English and French only. In addition, the abstract or meeting articles as well as congress posters were excluded although their scientific contribution could have been taken into consideration. This systematic review has not been registered online. Bias due to selective non-reporting (or incomplete reporting) that were measured and analyzed by the trial investigators are likely not to be disclosed. This literature review and data collected from EPAR are biased to published data that may not reflect the actual knowledge on the environmental impact of ATMP. Beyond existing undisclosed data, key considerations, such as dispersal of GMO from patient excreta in the clinical trials may not be known or planned in clinical trial development.

## Conclusion

Many challenges remain to be fulfilled in environmental contamination assessment related to the use of ATMP within the pharmacy preparation units, healthcare settings, and beyond. Because the use of these new treatments is a rapidly expanding field with increasing use in the clinical trials and routine practice, the guidelines are eagerly awaited. Even though environmental contamination assessment is poorly addressed in original articles related to the use of ATMP, most of the product information sections from EPAR suggested precautions rather than requirements when dealing with environmental consideration following ATMP handling. Nevertheless, information usually remains elusive especially concerning waste disposal and the detection of biological material on work surfaces, and mainly relate to the GMO than non-GMO cellular products. Pharmaceutical oversight and adherence to good preparation practices and good clinical practices are essential to ensure the safe use of these new therapeutics in healthcare setting in term of environmental concern. Additionally, this work demonstrates the necessity to adopt a multidisciplinary approach involving the clinicians, nurses, pharmacists, and biologists to assess and control environmental exposure to ATMP in the healthcare settings at all steps, from their reception to their administration, and suggest the importance to monitor excreta of a patient during the clinical trials to define recommendations to prevent environmental shedding following their use.

## Data Availability Statement

The original contributions presented in the study are included in the article/Supplementary Materials, further inquiries can be directed to the corresponding author/s.

## Author Contributions

MK and IM: Conceptualization. MK and AL: Methodology. MD, CF-L, and MK: Writing. MK, MD, and AL: Literature search. MD, CF-L, AL, SF, A-LC, SL, and VN: Critically revised work. MK: Supervision. All authors were involved in the design, data collection, analysis, and manuscript preparation.

## Funding

This work was supported by the Region Bourgogne Franche-Comté (PERSONALISE, subvention #2019-0077).

## Conflict of Interest

The authors declare that the research was conducted in the absence of any commercial or financial relationships that could be construed as a potential conflict of interest.

## Publisher's Note

All claims expressed in this article are solely those of the authors and do not necessarily represent those of their affiliated organizations, or those of the publisher, the editors and the reviewers. Any product that may be evaluated in this article, or claim that may be made by its manufacturer, is not guaranteed or endorsed by the publisher.

## Abbreviations

ATMP: advanced therapy medicinal products
CGT: cellular and gene therapy
EMA: European Medicines Agency
EPAR: European Public Assessment Reports
FDA: Food and Drug Administration
GMO: genetically modified organisms
PCR: polymerase chain reaction
SOP: standard operation procedure
SPC: summary of product characteristics
T-VEC: Talimogene laherparep VEC.
